# Hsp90AB1在非小细胞肺癌中高表达并且与肺腺癌患者不良预后相关

**DOI:** 10.3779/j.issn.1009-3419.2016.02.02

**Published:** 2016-02-20

**Authors:** 明慧 王, 林 冯, 萍 李, 廼珺 韩, 燕宁 高, 汀 肖

**Affiliations:** 100021 北京，北京协和医学院，中国医学科学院肿瘤医院，分子肿瘤学国家重点实验室 癌发生及预防分子机理北京市重点实验室 State Key Laboratory of Molecular Oncology, Beijing Key Laboratory for Carcinogenesis and Cancer Prevention, Cancer Institute (Hospital), Peking Union Medical College & Chinese Academy of Medical Sciences, Beijing 100021, China

**Keywords:** Hsp90AB1, 免疫组织化学, 肺腺癌, 预后, Hsp90AB1, Immunohistochemistry, Lung adenocarcinoma, Prognosis

## Abstract

**背景与目的:**

热休克蛋白90AB1（heat shock protein 90 kDa alpha, class B member 1, Hsp90AB1）是ATP依赖的高度保守的分子伴侣，在多种肿瘤细胞中过表达。在肿瘤发生发展的信号传导通路中起着重要作用的一些分子，如表皮生长因子受体（epidermal growth factor receptor, EGFR）、人类表皮生长因子受体-2（human epidermal growth factor receptor-2, HER2）等，均是Hsp90AB1的底物蛋白。Hsp90AB1与这些底物蛋白相互作用并参与细胞的多种病理生理过程。本研究通过检测Hsp90AB1在非小细胞肺癌（non-small cell lung cancer, NSCLC）组织中的蛋白表达情况以初步探讨其临床意义。

**方法:**

采用组织微阵列和免疫组织化学染色的方法检测Hsp90AB1在213例NSCLC及相应癌旁正常肺组织中的蛋白表达，并分析Hsp90AB1的表达与NSCLC临床病理参数及患者预后的关系。

**结果:**

Hsp90AB1在肺癌组织中的表达水平（阳性率54.0%）高于在正常肺组织中的表达水平（阳性率0.0%，*P* < 0.001）。Hsp90AB1在肺腺癌中的表达阳性率为61.2%，高于肺鳞癌组织37.9%（*P*=0.002），并且其高表达与肺腺癌患者的不良预后相关（*P*=0.032）。Hsp90AB1蛋白表达水平与临床分期、淋巴结转移、病理分级等因素无关（*P* > 0.05）。

**结论:**

Hsp90AB1在NSCLC组织中高表达，并且其表达水平与肺癌的病理类型及肺腺癌患者总生存期相关。

## Abstract (en)

**Background and objective:**

Heat shock protein 90 kDa alpha, class B member 1 (Hsp90AB1) is highly conserved ATP-dependent molecular chaperone, and over-expressed in a variety of tumor cells. Some molecules that play important roles in tumor development signaling pathways such as epidermal growth factor receptor (EGFR), human epidermal growth factor receptor-2 (HER2) are Hsp90AB1 client proteins. Hsp90AB1 interact with these client proteins and participate in a variety of pathophysiological processes of cells. The aim of this study is to detect the expression of Hsp90AB1 in non-small cell lung cancer (NSCLC) tissues, and explore its clinical significance.

**Methods:**

The expression of Hsp90AB1 in 213 NSCLC tissues and 147 normal lung tissues was detected by tissue microarray and immunohistochemical staining method, and the relationship of Hsp90AB1 expression with clinicopathological parameters and prognosis of NSCLC patients were analyzed.

**Results:**

The expression level of Hsp90AB1 in lung cancer tissues (positive rate of 54.0%) was significantly higher than that in normal lung tissue (positive rate of 0.0%, *P* < 0.001). The positive expression rate of Hsp90AB1 in lung adenocarcinoma tissues (61.2%) was significantly higher than that in lung squamous cell carcinoma tissues (37.9%)(*P*=0.002), and its over-expression was associated with poor prognosis in lung adenocarcinoma patients (*P*=0.032). The expression level of Hsp90AB1 had no significant correlation with clinical stage, lymph node metastasis, pathological grade or other factors (*P* > 0.05).

**Conclusion:**

Hsp90AB1 protein was over-expressed in NSCLC tissues, and was associated with lung cancer pathological type and overall survival in lung adenocarcinoma patients.

热休克蛋白90（heat shock protein, Hsp90）是ATP依赖的高度保守的分子伴侣，其可利用ATP水解产生的能量参与蛋白质的正确折叠，稳定蛋白质结构，参与细胞信号转导激素应答及转录调控等反应以及肿瘤凋亡、增殖相关通路的调节^[1, 2]^。Hsp90对因环境改变等各种应激状态下蛋白质稳定性的保持至关重要^[1]^。除了在正常细胞中发挥重要作用外，Hsp90与底物蛋白相结合参与多种疾病的发生发展，如恶性肿瘤、自身免疫性疾病。他还是多种致癌蛋白的分子伴侣，包括表皮生长因子受体（epidermal growth factor receptor, EGFR）、人类表皮生长因子受体-2（human epidermal growth factor receptor-2, HER2）、间质表皮转化因子（mesenchymal-epithelial transition, MET）、蛋白激酶B（protein kinase B, AKT）等^[3]^。目前认为，人类Hsp90主要包括4种亚型：Hsp90AA1和Hsp90AB1均位于胞质内，Grp94位于内质网中，而TRAP1亚型则定位于线粒体基质内。Hsp90AA1和Hsp90AB1有85%同源，但是两者作用不同，Hsp90AA1是诱导性表达，参与应激状态下细胞保护和细胞周期的调节，而Hsp90AB1是组成性表达，参与早期胚胎发育、信号转导及细胞长期适应性^[4]^。

目前在世界范围内，肺癌发生率和死亡率均居恶性肿瘤首位，其中约85%为非小细胞肺癌^[5]^。作为分子伴侣，Hsp90有助于多种促肺癌蛋白质的折叠和成熟，例如EGFR和ALK。阻断这些伴侣蛋白发挥功能是癌症治疗中的一种全新策略。Ramalingam等^[6]^对Hsp90新型抑制剂ganetespib的一项临床Ⅱ期研究发现，在治疗晚期肺腺癌患者过程中，ganetespib与多西他赛（docetaxel）联合用药治疗与多西他赛单药治疗相比，可延长晚期肺腺癌患者的总生存期。本研究采用免疫组织化学染色方法检测Hsp90AB1在213例非小细胞肺癌组织中的蛋白表达水平，分析其与非小细胞肺癌临床病理参数及患者预后的关系，为Hsp90抑制剂在肺腺癌患者的临床用药提供理论依据。

## 1 材料和方法

### 1.1 标本

肺癌组织微阵列：上海芯超生物科技有限公司，（HLug-Scc150Squ-01, HLug-Ade150Sur-01），手术时间2004年7月-2007年11月，随访时间截止至2012年7月；上海卓立生物科技有限公司（LUC1505），手术时间2005年7月-2011年12月，随访时间截止至2013年6月。三张组织芯片共包含213例肺癌组织和相应的癌旁肺组织（距离肿瘤组织3 cm以外的正常肺组织），其中肺鳞癌66例，肺腺癌147例。

### 1.2 免疫组化试剂

一抗为Hsp90AB1兔多克隆抗体（货号：AP7867d，Abgent，美国），工作浓度是1:100；二抗为山羊抗兔IgG/HRP（中杉金桥，中国）；30%H_2_O_2_溶液（西陇化工，中国）；10×抗原修复液（柠檬酸盐缓冲液，0.01 M，pH6.0）（中杉金桥，中国）；正常山羊血清（中杉金桥，中国）；20×DAB显色试剂盒（中杉金桥，中国）；苏木素染料（中杉金桥，中国）。

### 1.3 免疫组化实验步骤（SP法）

组织芯片置于65 ℃烤箱中，烘烤3 h；经脱蜡、水化后放置于3%的H_2_O_2_溶液中室温避光孵育10 min，以除去细胞内源性过氧化物酶的活性；抗原修复液进行抗原修复；正常山羊血清封闭液室温封闭10 min；弃封闭液，用一抗工作液（Hsp90AB1兔多克隆抗体，1:100）4 ℃孵育过夜；滴加生物素化的二抗工作液（山羊抗兔IgG），室温孵育30 min；DAB显色，显微镜下控制染色强度；苏木素染色；树脂封片，显微镜下观察^[7, 8]^。

### 1.4 结果判定

免疫组化染色结果由两位独立临床病理医师阅片判断。根据细胞染色强度评分和阳性细胞比例评分的乘积进行半定量分析。按细胞染色强度评分：0分（无棕黄色颗粒），1分（淡黄色颗粒），2分（棕黄色颗粒），3分（褐色颗粒）；按阳性细胞比例评分：0分（阳性细胞比例≤5%），1分（5% < 阳性细胞比例≤25%），2分（25% < 阳性细胞比例≤50%），3分（50% < 阳性细胞比例≤75%），4分（75% < 阳性细胞比例≤100%）。结果取两者之积，分为4级：0记为（–），1-4记为（+），5-8记为（++），9-12记为（+++）。将（-）和（+）定为蛋白表达阴性，将（++）和（+++）定为蛋白表达阳性。

### 1.5 统计学方法

采用SPSS 17.0（SPSS INC，美国）和SigmaPlot 12.3（Systat Software，San Jose，美国）软件进行数据统计分析。生存期计算从手术日期开始到随访日期终止，或由于复发、转移而死亡的日期或失访日期终止。采用卡方检验分析肺癌中Hsp90AB1的表达与各临床参数之间的关系，采用*Kaplan*-*Meier*计算肺腺癌患者的生存曲线，并进行对数秩检验，用*Cox*多因素回归分析各种因素对肺腺癌患者总生存期的影响。*P* < 0.05为差异具有统计学意义。

## 2 结果

### 2.1 Hsp90AB1在非小细胞肺癌组织中高表达

免疫组化结果显示Hsp90AB1阳性表达部位主要是肺癌细胞的胞浆，呈棕黄色颗粒状弥漫分布在肺癌细胞胞浆中（图 1，图 2B、图 2C）。免疫组化检测213例非小细胞肺癌，其中115例肺癌组织中Hsp90AB1呈阳性表达，阳性率为54.0%。Hsp90AB1在正常癌旁肺组织中几乎不表达（图 2A）。

**1:**
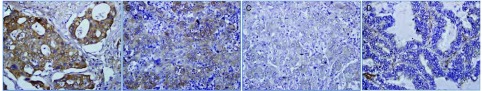
运用Hsp90AB1兔多克隆抗体在组织芯片中用免疫组织化学染色方法检测Hsp90AB1在肺腺癌组织中的表达（×400）。A-D分别表示Hsp90AB1在肺腺癌组织中的染色强度为（+++）、（++）、（+）、（-）。 Hsp90AB1 protein expression in lung adenocarcinoma tissues, as observed by immunohistochemical staining with a rabbit polyclonal antibody against Hsp90AB1, applied to formalin-fixed and paraffin-embedded tissues in the manner of tissue microarrays (Original magnification: ×400). A-D: The expression intensities of Hsp90AB1 immunostaining in lung adenocarcinoma tissues were (+++), (++), (+), (-), respectively. Hsp90AB1: heat shock protein 90 kDa alpha, class B member 1.

**2:**
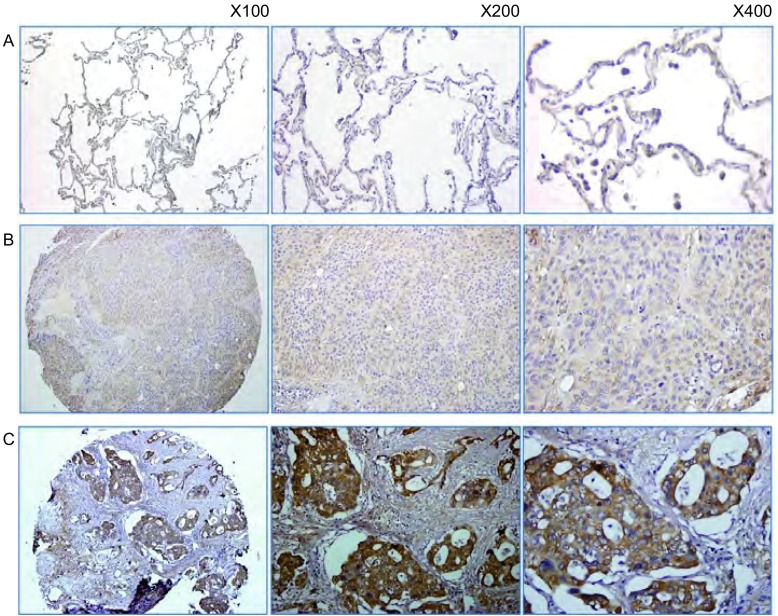
运用Hsp90AB1兔多克隆抗体在组织芯片中用免疫组织化学染色方法检测Hsp90AB1在正常肺组织和肺癌组织中的表达。A：Hsp90AB1在癌旁正常肺组织中不表达（×100）；B：Hsp90AB1在肺鳞癌组织中低表达（×200）；C：Hsp90AB1在肺腺癌组织中高表达（×400）。 Hsp90AB1 protein expression in normal lung tissues and lung cancer tissues, as observed by immunohistochemical staining with a rabbit polyclonal antibody against Hsp90AB1, applied to formalin-fixed and paraffin-embedded tissues in manner of tissue microarrays. A: Hsp90AB1 was not expressed in normal lung tissues (×100); B: Low expression of Hsp90AB1 in lung squamous cell carcinoma tissues (×200); C: High expression of Hsp90AB1 in lung adenocarcinoma tissues (×400).

### 2.2 Hsp90AB1表达与肺癌病理类型相关

213例肺癌组织中，有147例肺腺癌，66肺鳞癌，其中Hsp90AB1在肺鳞癌中的阳性表达率为37.9%，在肺腺癌中的阳性表达率为61.2%（表 1，图 2B、图 2C），差异有统计学意义（*P*=0.002）。Hsp90AB1在非小细胞肺癌组织中的表达与肿瘤的临床分期、淋巴结转移、病理分级等因素无相关性（*P* > 0.05）。Hsp90AB1的表达水平与各临床参数之间的关系总结见表 1。

**1:** 肺癌中Hsp90AB1的表达与临床病理参数之间的关系 Correlation between clinicopathological features and Hsp90AB1 expression in lung cancer

Clinical parameters	*n*	Intensity (%)		*P*
		-/+	++/+++	
Tissues				< 0.001
Normal tissues	213	213 (100.0)	0 (0.0)	
Primary tumors	213	98 (46.0)	115 (54.0)	
Age (year)				0.531
> 60	107	50 (46.7)	57 (53.3)	
≤60	106	48 (45.3)	58 (54.7)	
Gender				0.174
Male	142	70 (49.3)	72 (50.7)	
Female	71	28 (39.4)	43 (60.6)	
Pathological grade				0.413
High	40	20 (50.0)	20 (50.0)	
Middle	106	51 (48.1)	55 (51.9)	
Low	62	24 (38.7)	38 (61.3)	
Missing	5	-	-	
Clinical stage				0.167
Ⅰ+Ⅱ	153	67 (43.8)	86 (56.2)	
Ⅲ+Ⅳ	49	27 (55.1)	22 (44.9)	
Missing	11	-	-	
Pathological type				0.002
SCC	66	41 (62.1)	25 (37.9)	
ADC	147	57 (38.8)	90 (61.2)	
Lymph node metastasis				0.603
Yes	87	42 (48.3)	45 (51.7)	
No	121	54 (44.6)	67 (55.4)	
Missing	5	-	-	
*P*-value from pearson’s *χ*^2^ test; ADC: adenocarcinoma; SCC: squamous cell carcinoma.				

### 2.3 Hsp90AB1表达与肺腺癌预后相关

本研究中上海芯超和上海卓立科技有限公司的两张组织芯片共包含147例肺腺癌组织，两个公司的组织芯片构成病例在男女性别，临床分期、分化程度等临床参数的组成比例无明显差别。随访时间0.06年至8.0年，中位随访时间3.4年。Hsp90AB1阳性表达的肺腺癌患者总生存率低于Hsp90AB1阴性表达的患者（*P*=0.032，图 3）。*Cox*多因素回归分析结果显示，Hsp90AB1可以作为预后的独立因素（表 2，*P*=0.002）。

**2:** 肺腺癌患者总生存期的*Cox*多因素回归分析 Multivariate *Cox* regression analysis for overall survival in lung adenocarcinoma patients

Variable	Overall survival (*n*=147)	
	Hazard ratios (95%CI)	*P*
Age (year)		
> 60/≤60	0.908 (0.536-1.538)	0.719
Gender		
Male/Female	1.004 (0.606-1.664)	0.987
Pathological grade		
High/Others	0.850 (0.461-1.566)	0.602
Clinical stage		
Ⅰ+Ⅱ/Ⅲ+Ⅳ	1.904 (0.627-5.780)	0.256
Lymph node metastasis		
Yes/No	1.436 (0.813-2.536)	0.212
Hsp90AB1		
Positive/Negative	0.448 (0.269-0.745)	0.002

**3:**
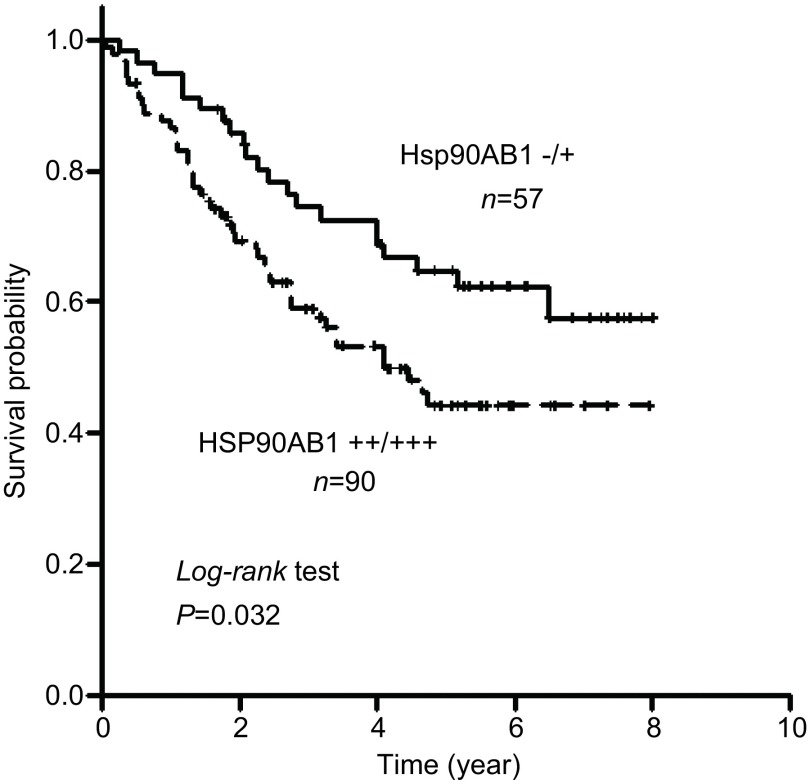
*Kaplan*-*Meier*生存分析显示Hsp90AB1阳性组（*n*=90）肺腺癌患者比阴性组（*n*=57）预后差，横坐标表示患者的生存时间，纵坐标表示累计生存率。 The *Kaplan*-*Meier* survival analysis revealed that the lung adenocarcinoma patients with Hsp90AB1 positive expression (*n*=90) had a significantly poorer outcome than those with Hsp90AB1 negative expression (*n*=57)(*P*=0.032). The abscissa represents the patient's survival time and the vertical axis indicates survival probability.

## 3 讨论

目前已经发现超过200种蛋白与Hsp90相互作用成为其底物蛋白，大多数的这些底物蛋白参与必要的细胞增殖，细胞周期进程和细胞凋亡调控，包括类固醇激素受体、激酶、转录因子、HER2、Akt、突变型P53等^[9, 10]^。Hsp90在调节底物蛋白构象的正确折叠、稳定性及功能方面具有核心作用^[11]^。Hsp90底物蛋白有多种是致癌蛋白，其对致癌蛋白的稳定性和功能的发挥具有关键作用。肿瘤的一些特征性变化都与这些致癌蛋白相关，如持续的抗凋亡、血管生成及组织浸润和转移^[12]^。

目前在世界范围内肺癌是死亡率最高的恶性肿瘤，占癌症死亡总数的三分之一，非小细胞肺癌占85%的肺癌病例，其中肺腺癌是非小细胞肺癌的主要组织学类型^[13]^。目前关于Hsp90在肺癌组织中蛋白表达情况的研究较少。我们研究发现，Hsp90AB1在肺腺癌组织中的阳性表达率为61.2%，高于在肺鳞癌中的表达。Hsp90AB1在肺腺癌组织中表达高的原因之一是*EGFR*在肺腺癌中的突变率高，而EGFR是Hsp90AB1的一种底物蛋白，据相关研究^[14]^显示EGFR在白种人群肺腺癌中的突变率约为15%-20%，在亚洲人群肺腺癌中的突变率高达50%。此外我们的研究结果还显示Hsp90AB1的高表达与肺腺癌的不良预后相关，*Cox*多因素回归分析表明Hsp90AB1可作为影响肺腺癌预后的独立危险因素。此结果与文献报道的Hsp90在其他肿瘤的表达情况一致，Wang等^[15]^通过免疫组化法在322例胃癌样本中研究结果显示Hsp90与胃癌的侵袭、转移及预后相关。Shirota等^[16]^研究发现Hsp90在胆管癌中的表达与胆管癌的5年生存率明显相关。此外，也有研究^[17, 18]^发现Hsp90与乳腺癌的预后相关。这些研究结果提示高表达Hsp90可以作为判断某些癌症患者预后的标志物，为肿瘤患者手术后的治疗提供参考。

Hsp90能够帮助癌细胞克服各种不良应激刺激，如缺氧、蛋白质毒性压力以及营养物质缺乏等，并且他还能保护癌细胞逃避免疫系统的攻击^[19, 20]^。正是因为Hsp90的这一特点使得其成为癌症治疗的一个极具价值的靶点。目前针对Hsp90抑制剂的研究较多，Ueno等^[21]^在研究AUY922对几种已发生遗传改变，包括*EGFR*突变的非小细胞肺癌细胞系的抗肿瘤实验结果表明，AUY922不仅在*EGFR*突变的肿瘤，而且在非*EGFR*突变但含有*K*-*ras*突变或*EML*-*ALK*基因融合等分子改变的肿瘤细胞中抗肿瘤作用明显增强。并且其在胆管癌细胞中也发挥着抗生长和抗血管生成的作用^[15]^。所以，AUY922有望成为新型抗肿瘤药物。Gomez-Casal等^[22]^研究Hsp90另一种抑制剂Ganetespib发现其可以抑制肺腺癌细胞增殖、迁移，诱导肺腺癌细胞凋亡，并且可以增加肺腺癌细胞体外放射敏感性。本研究的结果表明Hsp90AB1在肺腺癌组织中表达阳性率为61.2%，且这部分Hsp90AB1高表达的患者预后较差。如果针对Hsp90阳性表达的患者使用Hsp90抑制剂将更有针对性，提高用药的有效率。

综上所述，肺癌组织中Hsp90AB1高表达，尤其在肺腺癌组织中阳性表达是患者预后的独立因素，为临床上使用Hsp90抑制剂作为肺腺癌靶向药物提供理论基础，更能针对性用药。
